# Quantifying activities of daily living impairment in Parkinson’s disease using the Functional Activities Questionnaire

**DOI:** 10.1007/s10072-021-05365-1

**Published:** 2021-06-10

**Authors:** Sara Becker, Claire Pauly, Michael Lawton, Geraldine Hipp, Francesca Bowring, Patricia Sulzer, Michele Hu, Rejko Krüger, Thomas Gasser, Inga Liepelt-Scarfone

**Affiliations:** 1 Department of Neurodegenerative Diseases, Hertie Institute for Clinical Brain Research, Hoppe-Seyler-Str. 3, 72076 Tübingen, Germany; 2 German Center for Neurodegenerative Diseases, Tübingen, Germany; 3 Clinical and Experimental Neuroscience, Luxembourg Centre for Systems Biomedicine (LCSB), University of Luxembourg, Luxembourg, Luxembourg; 4 Parkinson’s Research Clinic, Centre Hospitalier de Luxembourg (CHL), Luxembourg, Luxembourg; 5 Department of Population Health Sciences, University of Bristol, Bristol, UK; 6 Nuffield Department of Clinical Neurosciences, Division of Clinical Neurology, University of Oxford, Oxford, UK; 7 Oxford Parkinson’s Disease Centre, University of Oxford, Oxford, UK; 8 Transversal Translational Medicine, Luxembourg Institute of Health (LIH), Strassen, Luxembourg; 9 Studienzentrum Stuttgart, IB Hochschule, Stuttgart, Germany

**Keywords:** Cognitive dysfunction, Cohort studies, Mild cognitive impairment, Neuropsychological tests

## Abstract

**Objective:**

Cognitive-driven activity of daily living (ADL) impairment in Parkinson’s disease (PD) is increasingly discussed as prodromal marker for dementia. Diagnostic properties of assessments for this specific ADL impairment are sparsely investigated in PD. The ability of the Functional Activities Questionnaire (FAQ) for differentiating between PD patients with normal cognition and with mild cognitive impairment (PD-MCI), according to informant and self-reports, was examined. Global cognitive function in groups with and without mild ADL impairment was compared according to different cut-offs.

**Methods:**

Multicenter data of 589 patients of an international cohort (CENTRE-PD) were analyzed. Analyses were run separately for informant-rated and self-rated FAQ. Receiver operating characteristic (ROC) analysis was conducted to define the optimal FAQ cut-off for PD-MCI (≥ 1), and groups were additionally split according to reported FAQ cut-offs for PD-MCI in the literature (≥ 3, ≥ 5). Binary logistic regressions examined the effect of the Montreal Cognitive Assessment (MoCA) score in PD patients with and without mild ADL impairment.

**Results:**

Two hundred and twenty-five (38.2%) patients were classified as PD-MCI. For all three cut-off values, sensitivity was moderate to low (< 0.55), but specificity was moderately high (> 0.54) with a tendency of higher values for self-reported deficits. For the self-report, the cut-off ≥ 3 showed a significant effect of the MoCA (*B* =  − 0.31, *p* = 0.003), where FAQ ≥ 3 patients had worse cognition. No effect for group differences based on informant ratings was detected.

**Conclusion:**

Our data argue that self-reported ADL impairments assessed by the FAQ show a relation to the severity of cognitive impairment in PD.

**Supplementary Information:**

The online version contains supplementary material available at 10.1007/s10072-021-05365-1.

## Introduction

Mild cognitive impairment (MCI) in Parkinson’s disease (PD-MCI) has been defined as a prodromal stage of Parkinson’s disease (PD) dementia (PDD) [1]. While PD-MCI patients are at greater risk of developing PDD [2], it is not possible to predict which patients convert within a short time period. Additional markers indicating patients at risk for PDD conversion are therefore urgently needed.

In non-PD cohorts, instrumental activity of daily living (ADL) impairments have been reported to emerge in the transition from MCI to dementia, affecting complex skills such as managing finances [3, 4]. Impairments in these functional abilities are even strong predictors of future conversion to dementia [5]. In PD, research demonstrates that non-demented patients already show functional impairment [6, 7], primarily related to loss of cognitive function [8, 9]. Mild ADL impairment in PD-MCI patients might help to identify those at risk for PDD conversion [7, 8], necessitating an early diagnosis of ADL impairment in the prodromal stage of PDD using sensitive and reliable measures.

One of the most commonly used scales to measure ADL impairment is the Pfeffer Functional Activities Questionnaire (FAQ) [10], which has a high discriminative ability for distinguishing MCI from other diagnostic groups [11]. For non-PD cohorts, a cut-off score of ≥ 5 points has been discussed to define MCI [10, 12], yet only very few studies have examined cut-off values for PD-MCI. A recent paper examining both Alzheimer’s disease and PD patients found that an FAQ score ≥ 3 was able to differentiate cognitively normal PD (PD-CN) and PD-MCI patients, after matching for age, sex, and education [13]. They found a low sensitivity (38.1%) but high specificity (92.9%), attributing this to the fact that only a subgroup of PD-MCI patients presented with ADL dysfunction. A notable limitation of this study was that they did not examine the cognitive profile of PD patients stratified according to this cut-off. Standardized ADL assessments accompanied by neuropsychological testing can further the understanding of how cognitive abilities are related to ADL impairment [14], which is necessary to identify measures that are sensitive to early changes in functional abilities.

The FAQ is most commonly completed by an informant, as statements by relatives about the patient ‘s ADL situation are often of great importance for further clinical decisions. It can also be completed by the patient themselves if no caregiver is available, to provide insight into how cognitive impairment affects their daily life. Previous research has shown that PD patients tend to underestimate their ADL impairment compared to their caregivers [14, 15]. However, not all studies have found differences between informant and self-reporting of ADL function in PD [16, 17]. It is still unclear whether self-reports or informant reports are more useful in the clinical routine for judging cognitive-driven ADL deficits, as the discrepancy between the two types of reports have not been extensively studied.

The aim of this study was to examine the diagnostic abilities of different cut-off scores of the FAQ for differentiating between levels of severity of cognitive impairment in PD, differentiating between informant and self-reports. For this purpose, diagnostic values of cut-offs reported in the literature and the optimal cut-off derived from the current data were examined in an international pooled cohort of patients. Global cognitive function was also compared between groups for each cut-off, to determine how cognition relates to daily functioning in PD.

## Methods

### Study design and participants

Data of 679 patients were harmonized and analyzed within the frame of the CENTRE-PD project. Patients were recruited from the ABC-PD Study at the University Hospital in Tübingen (*n* = 226) [8], the Luxembourg Parkinson’s Study at the University of Luxembourg (*n* = 274) [18], and the OPDC Discovery Cohort at the University of Oxford (*n* = 179) [19]. All studies received ethical approval from local ethics committees; all patients gave written informed consent for respective study participation at each of the three study centers. For all studies, procedures were in accordance with the 1964 Helsinki declaration and its later amendments or comparable ethical standards.

Inclusion criteria for the analyses were age between 45 and 90 years, and ability to give informed consent. Patients who met the following criteria were excluded: severe cognitive impairment determined by a Montreal Cognitive Assessment (MoCA) score < 18 points (*n* = 14, 2.1%), major depression defined by a Beck Depression Inventory (BDI)-II score > 19 points after conversion (44, 6.5%,), or missing demographic, FAQ, or MoCA data (32, 4.7%). Data of 589 patients was included in the final data set.

### Assessments

Demographics were collected for each patient, and the Unified Parkinson’s Disease Rating Scale-Part III (UPDRS-III) and the Hoehn and Yahr staging scale assessed motor severity [20]. Depressive symptomatology was assessed using the BDI versions I (BDI-I) [21] and II (BDI-II). All BDI-I values were converted to the appropriate BDI-II scores according to the BDI-II manual. The MoCA was used to assess global cognitive functioning [22]. According to Level-I criteria of the Movement Disorders Society [23], patients were classified as PD-CN if they had an MoCA score ≥ 26, or as PD-MCI if they scored between 18 and 25 points. The FAQ, which consists of ten items, each rated from 0 (normal) to 3 (dependent), was used to assess ADL impairment. The total score ranges from 0 to 30 points, with higher scores suggesting greater functional dependence.

### Statistical analyses

SPSS version 26 (SPSS Inc., Chicago, IL, USA) was used to conduct statistical analyses, with α levels set at 0.05. The Shapiro–Wilk test was applied to test for normal distribution of variables. Demographics were compared between cognitive groups (PD-CN and PD-MCI) using Chi-squared and Mann–Whitney-*U* tests where appropriate. A receiver operating characteristic (ROC) analysis was conducted to define the optimal (highest Youden’s Index) FAQ cut-off to define PD-MCI. The FAQ cut-offs chosen for the following analyses were (i) optimal cut-off identified from the ROC curve analysis, (ii) cut-off ≥ 3 shown to differentiate between PD-CN and PD-MCI [13], and (iii) a cut-off for MCI (≥ 5) in the general population [10, 12]. Sensitivity and specificity as well as the positive predictive value (PPV) and negative predictive value (NPV) were calculated for each of the three cuts-offs. This was done separately for informant-rated FAQ and self-rated FAQ to compare the diagnostic abilities of both ratings. Binary logistic regressions examining the effect of the MoCA total score between each FAQ cut-off group while correcting for significant demographic variables were conducted in the informant-rated and self-rated FAQ groups separately. As the FAQ has been found to be dependent on age in older adults [24], we chose to include age as a constant covariate, even if it was not significantly different between groups. It is also important to note that PD is a neurodegenerative disease, and increasing ADL and cognitive impairment has been shown to be associated with motor symptom worsening and higher PD severity [25]. Therefore, disease duration was also included as a covariate in all models.

## Results

Of all patients, 225 (38.2%) were classified as PD-MCI and 364 (61.8%) as PD-CN. PD-MCI patients were significantly older, had less formal education, had greater severity of motor symptoms, and showed more impairment with ADL activities than PD-CN (see Table 1). The FAQ was most commonly completed by the patient’s spouse (419, 71.1%), followed by the patient themselves (136, 23.1%), the patient’s child (22, 3.7%), a close friend (7, 1.2%), or a non-specified informant (5, 0.8%).

**Table 1:** Demographic and clinical characteristics of the pooled sample

	Total sample *N* = 589	PD–CN *n* = 364	PD–MCI *n* = 225	*p*-value
Male sex: n (%)	388 (65.9)	224 (61.5)	164 (72.9)	**0.006**
Age (years)	68.47 (46.52–89.93)	65.29 (46.52–89.93)	72.30 (47.47–89.28)	** < 0.001**
Education years	13 (5–31)	14 (5–25)	12 (5–31)	** < 0.001**
Disease duration years	5 (0–31)	4.89 (0–26)	5.36 (0–31)	0.37
UPDRS-III total score	30 (1–81)	28 (1–79)	33 (3–81)	** < 0.001**
Hoehn and Yahr: n (%)				** < 0.001**
1	49 (8.3)	38 (10.4)	11 (4.9)	
2	387 (65.7)	252 (69.3)	135 (60)	
3	137 (23.3)	68 (18.7)	69 (30.7)	
4	16 (2.7)	6 (1.6)	10 (4.4)	
BDI-II total score	7 (0–19)	7 (0–19)	8 (0–19)	0.56
FAQ total score	0 (0–29)	0 (0–21)	2 (0–29)	** < 0.001**
MoCA total score	26 (18–30)	28 (26–30)	23 (18–25)	–*

Results are expressed as median (range) except where noted; boldface indicates statistically significant values

*BDI-II* Beck Depression Inventory-II; *FAQ* Functional Activities Questionnaire; *MoCA* Montreal Cognitive Assessment; *PD-CN* Parkinson’s Disease with normal cognition; *PD-MCI* Parkinson’s disease with mild cognitive impairment; *UPDRS-III* Unified Parkinson’s Disease Rating Scale-Part III

^*^Variable was used to split groups, statistical comparison not possible

The ROC curve analysis of the FAQ total score for diagnosing PD-MCI produced an area under the curve of 0.61 and standard error 0.02, *p* < 0.001 (95% confidence interval: 0.56–0.66), which was judged to be sufficient [26]. Diagnostic values for differentiating PD-CN from PD-MCI can be found in Table 2 for all three cut-offs. In all patients, 132 (58.7%) of PD-MCI compared to 156 (42.9%) PD-CN patients scored ≥ 1 on the FAQ. Eighty-eight (39.1%) PD-MCI compared to 78 (21.4%) PD-CN patients scored above the cut-off of 3. For FAQ ≥ 5, 57 (25.3%) PD-MCI compared to only 51 (14%) of PD-CN patients scored above the cut-off.

**Table 2:** Diagnostic values of the FAQ total score for differentiating between PD-CN and PD-MCI according to each chosen cut-off value

FAQ cut-off	Sensitivity	Specificity	PPV	NPV
All patients				
≤ 1	0.59	0.57	0.46	0.69
≤ 3	0.39	0.79	0.53	0.68
≤ 5	0.25	0.86	0.53	0.65
Informant ratings				
≤ 1	0.54	0.67	0.52	0.69
≤ 3	0.32	0.90	0.68	0.68
≤ 5	0.22	0.95	0.75	0.65
Self ratings				
≤ 1	0.60	0.54	0.44	0.69
≤ 3	0.42	0.75	0.50	0.68
≤ 5	0.26	0.83	0.49	0.65

*FAQ* Functional Activities Questionnaire; *NPV* negative predictive value; *PPV* positive predictive value

### Informant analyses

Demographics for groups split according to each of the three cut-offs are shown in Supplementary Table 1. For all cut-offs, significant differences were found for age, disease duration, UPDRS-III, BDI-II, and MoCA total scores, where patients scoring above each cut-off were older, had more severe motor impairment and longer disease durations, increased depressive symptomatology, and lowered cognition. There was a significant effect of sex for the cut-off of 1, with more males scoring above the cut-off. Additionally, patients scoring above the cut-off of 5 had significantly lower formal education years than those scoring below the cut-off. Diagnostic ability for differentiating PD-CN from PD-MCI for all three cut-off values can be found in Table 2.

Binary logistic regressions were used to examine the effect of the MoCA, age, and significant demographic variables on each FAQ cut-off (see Table 3). When correcting for sex, age, disease duration, UPDRS-III, and BDI-II, the regression model for FAQ cut-off of 1 [*χ*^*2*^(6) = 86.23, *p* < 0.001] was not predicted by the MoCA. Splitting the groups according to the FAQ cut-off 3 [model: *χ*^*2*^(5) = 96.88, *p* < 0.001], with age, disease duration, UPDRS-III, and the BDI-II as covariates, also did not reveal a significant effect of the MoCA. For the FAQ cut-off of 5 using age, education, disease duration, UPDRS-III, and the BDI-II as covariates [model fit: *χ*^*2*^(6) = 82.31, *p* < 0.001], the MoCA did not significantly differentiate between groups.

**Table 3:** Binary logistic regression models between Functional Activities Questionnaire Cut-offs and the Montreal Cognitive Assessment for Informant Ratings

Model	Predictors	Nagelkerke *R*^*2*^	*B*	*SE B*	*p*-value	Odds ratio	95% CI (lower–upper)
Cut-off 1		0.23					
	MoCA total score		− 0.02	0.04	0.64	0.98	0.91–1.06
	Sex		− 0.51	0.23	**0.02**	0.60	0.39–0.93
	Age		0.02	0.01	0.06	1.02	1.00–1.05
	Disease duration		0.02	0.03	0.40	1.02	0.97–1.07
	UPDRS-III		0.04	0.01	** < 0.001**	1.04	1.02–1.06
	BDI-II		0.12	0.02	** < 0.001**	1.13	1.08–1.17
Cut-off 3		0.27					
	MoCA		− 0.05	0.04	0.22	0.95	0.87–1.03
	Age		0.03	0.01	**0.04**	1.03	1.00–1.06
	Disease duration		0.05	0.03	**0.04**	1.06	1.00–1.11
	UPDRS-III		0.04	0.01	** < 0.001**	1.04	1.02–1.07
	BDI-II		0.12	0.02	** < 0.001**	1.12	1.07–1.18
Cut-off 5		0.26					
	MoCA		− 0.01	0.05	0.84	0.99	0.90–1.09
	Age		0.04	0.02	**0.01**	1.05	1.01–1.08
	Education years		− 0.06	0.04	0.10	0.94	0.87–1.01
	Disease duration		0.03	0.03	0.36	1.03	0.97–1.09
	UPDRS-III		0.05	0.01	** < 0.001**	1.05	1.03–107
	BDI-II		0.11	0.03	** < 0.001**	1.12	1.06–1.17

Boldface indicates statistically significant values

*B* unstandardized beta; *BDI-II* Beck Depression Inventory-II; *CI* confidence interval of the odds ratio; *MoCA* Montreal Cognitive Assessment; *SE B* standard error for unstandardized beta; *UPDRS-III*, Unified Parkinson’s Disease Rating Scale III

### Self-report analyses

Supplementary Table 2 shows the demographics for all three groups split according to the cut-offs. Significant effects were found for all three cut-offs for UPDRS-III and MoCA total scores, with patients scoring below each cut-off showing increased motor severity and lowered cognition. There was a significant effect of the BDI-II for cut-offs 3 and 5, with patients scoring above the cut-off displaying greater depressive symptoms than those below the cut-offs. Additionally, an effect of disease duration was found for the cut-off of 3, where patients scoring above the cut-off had longer disease durations than those below the cut-off. Table 2 shows the diagnostic values for differentiating PD-CN from PD-MCI for all three cut-offs.

Relation between FAQ groups per cut-off and the MoCA, including age and significant demographic variables as covariates, was examined using binary logistic regressions (see Table 4). The model using the FAQ cut-off of 1 and age, disease duration, UPDRS-III, and BDI-II as covariates was stable, *χ*^*2*^(4) = 15.97, *p* = 0.007, yet the MoCA was not able to differentiate between groups. Splitting the groups according to the FAQ cut-off 3 [model: *χ*^*2*^(5) = 35.49, *p* < 0.001] using age, disease duration, UPDRS-III, and the BDI-II as covariates showed a significant effect of the MoCA [*B* =  − 0.31, Exp(*B*) = 0.74, *p* = 0.003], where patients with an FAQ ≥ 3 had worse cognition. For the FAQ cut-off of 5 and correcting for age and UPDRS-III [model fit: *χ*^*2*^(3) = 20.74, *p* < 0.001], the MoCA showed moderate evidence for differentiating between groups, although this did not reach significance.

**Table 4:** Binary logistic regression models between Functional Activities Questionnaire Cut-offs and the Montreal Cognitive Assessment for Self Ratings

Model	Predictors	Nagelkerke *R*^*2*^	*B*	*SE B*	*p*-value	Odds ratio	95% CI (lower–upper)
Cut-off 1		0.15					
	MoCA total score		− 0.08	0.07	0.25	0.92	0.80–1.06
	Age		0.02	0.02	0.40	1.02	0.97–1.07
	Disease duration		0.03	0.05	0.55	1.03	0.93–1.15
	UPDRS-III		0.04	0.02	**0.02**	1.04	1.01–1.07
	BDI-II		0.04	0.04	0.31	1.04	0.96–1.13
Cut-off 3		0.37					
	MoCA		− 0.31	0.10	**0.003**	0.74	0.60–0.90
	Age		− 0.01	0.04	0.80	0.99	0.92–1.06
	Disease duration		0.09	0.08	0.27	1.09	0.94–1.27
	UPDRS-III		0.06	0.02	**0.01**	1.06	1.02–1.11
	BDI-II		0.12	0.06	**0.03**	1.13	1.01–1.26
Cut-off 5		0.28					
	MoCA		− 0.21	0.11	0.06	0.81	0.65–1.01
	Age		0.05	0.04	0.26	1.05	0.97–1.13
	Disease duration		0.06	0.08	0.49	1.06	0.90–1.24
	UPDRS-III		0.06	0.02	**0.01**	1.07	1.02–1.12

Boldface indicates statistically significant values

*B* unstandardized beta; *BDI-II* Beck Depression Inventory-II; *CI* confidence interval of the odds ratio; *MoCA* Montreal Cognitive Assessment; *SE B* standard error for unstandardized beta; *UPDRS-III* Unified Parkinson’s Disease Rating Scale III

## Discussion

Recent studies have shown that PD-MCI patients already show the first signs of ADL dysfunctions [6, 9]; however, the quantification of these deficits remains difficult. The aim of this study was to examine the diagnostic abilities of different cut-off scores of the FAQ (from the current data set as well as the literature) for differentiating not only between cognitive impairment levels but also between informant and self-reports of ADL abilities.

The current results demonstrate that with increasingly higher FAQ cut-offs, for both self and informant reports, specificity increases indicating a higher ability of the cut-off to correctly identify PD-CN patients without ADL impairment. However, sensitivity also decreases, leading to a decrease in true positive patients among the PD-MCI group. In the original study [10], the FAQ presented good sensitivity (0.85) and specificity (0.81) in terms of distinguishing normal healthy individuals and those with cognitive decline. In our international PD sample, we were able to replicate the specificity in both analyses for the cut-offs 3 and 5, yet the sensitivity was lacking for all (< 0.45). This finding of a low sensitivity of the FAQ compared to the higher specifity for ADL impairment in PD-MCI is similar to a recent study which found sensitivity and specificity (38.1% and 92.9%, respectively) using the FAQ cut-off ≥ 3 [13]. One reason for this effect could be that ADL impairment arises in the more advanced disease stage of PD-MCI and is limited to only a subgroup of PD-MCI patients. Mild ADL impairment primarily related to loss of cognitive function has been detected in ~ 30% of PD-MCI with various measurements [8, 9], supporting our hypothesis.

It should also be noted that assessing ADL impairments is difficult in PD, as motor impairments interfere with daily functioning [27]. The progression of motor dysfunction in PD, coupled with increasing age, leads to a poorer ability to execute ADL [28, 29] and a reduced health-related quality of life [30], while cognition and ADL function may decline in parallel [31, 32]. This may suggest that ADL functions simply decline with progression of the disease. However, a recent study examined both motor and cognitive influences on the FAQ in non-demented PD patients [8]. Results showed that PD-MCI patients had more cognitive-driven and motor-driven ADL impairments than PD-CN patients. As disease duration was not significant between the groups, it can be said that ADL impairments are more severe in PD-MCI patients irrespective of disease course. Future studies are needed to evaluate the predicitive value of mild ADL impairment in prodomal stage of PDD with the intention of further characterizing a high-risk group for conversion to dementia in PD and comparing between different types of PD-MCI.

We also investigated associations between the different FAQ cut-offs and the MoCA, a measure of global cognition. These associations were assessed separately for the FAQ rated by informants and the FAQ rated by the patient themselves. The accuracy of both patient and informant perceptions of ADL impairment, especially in the prodromal stage of PDD, has been sparsely studied. For the informant ratings, no FAQ cut-off was significantly associated with the MoCA after adjustment for other covariates. However, the cut-off of 3 in the self-report analyses was able to show a significant association with the MoCA, and the cut-off of 5 showed moderate evidence of an association which did not reach clinical significance. It should be noted that fewer patients scored above the cut-off of 5 than the cut-off of 3, which limits the statistical power in our sample. Our results are strengthened by other studies that have shown that patients can assess their own neuropsychological performance more accurately [33]. However, previous studies have found that informant reports are related to objective neuropsychological performance and more accurate than self-reports [17]. We could not replicate this by using the MoCA score as an external criteria to verify that the classified mild ADL impairment is related to cognitive dysfunction. Caregiver burden, including stress and depression, has been shown to affect informants’ ratings of functional abilities [34, 35]. As these were not measured in our study, it is possible that their influence on informant evaluations led to the null effects found in the current results. Moreover, informants might have the tendency to overestimate motor impairment on ADL performance [15], which could explain the lack of a significant effect on the MoCA score after diving the sample according to type of FAQ informant. Our current data indicate that self-reported ADL impairment assessed by the FAQ showed a relation to the severity of cognitive impairment in PD.

It is possible that ADL scales can serve as screening tools for clinicians to decide which further assessments, such as neuropsychological tests, are necessary [14]. The main advantage of the FAQ is that it is a short and easy to apply measure and could be administered quickly at patient visits by doctors to gauge functional abilities. However, due to the low specificities found for the different cut-offs used, it seems that the FAQ may not be useful as a stand-alone assessment for detecting cognitive impairment and may not offer a significant advantage compared to cognitive tests. We would argue that until a definitive cut-off value with adequate sensitivity and specificity can be identified for each cognitive group, the FAQ should be used in conjunction with neuropsychological tests, in line with the current recommendation of the Movement Disorders Society [23]. Perhaps the FAQ is more useful in detecting subtle changes in ADL function over time, even in different cognitive groups, which would make it an extremely valuable tool for tracking progression. Compared to healthy controls, PD patients are four times more likely to lose functional independence [36]. As this loss is irreversible, future studies should aim to improve the cut-off of the FAQ for identifying patients at risk of rapid decline in ADL function.

## Limitations

Some limitations need to be taken into account. The data analyzed were collected from different countries, raising the possibility of cross-cultural differences affecting the results; however, a previous study showed that the FAQ performed well across different ethnic groups [37]. As cognition is negatively influenced by severe depression [38], we excluded patients who showed signs of major depression which limits the generalization of our results to the larger population. Furthermore, to diagnose PD-MCI, only Level-I classification was used, instead of a comprehensive neuropsychological battery (Level-II), which may have led to an over-reporting of PD-MCI in our sample.

## Conclusion

This study has taken an important step towards determining a suitable cut-off for ADL dysfunctions in PD-MCI. Our data argue that the presence of mild ADL impairment in PD is associated with cognitive impairment. The utility of the FAQ for diagnosing PD-MCI may therefore be difficult due to the lack of specific criteria defining ADL impariment in PD-MCI. Future studies are needed to verify whether these patients are at greater risk for PDD conversion. If ADL function declines early in the disease course, and possibly reflects faster cognitive decline, it would be paramount to identify when to start treatments and to further research into examining what treatments can be developed.

## Supplementary Information

Below is the link to the electronic supplementary material.

Supplementary file1 (DOCX 20.0 KB)

### Supplementary Information

Below is the link to the electronic supplementary material.

Supplementary file2 (DOCX 16.0 KB)

## Data Availability

Not applicable.

## References

[1] Goldman JG, Litvan I (2011). Mild cognitive impairment in Parkinson's disease. Minerva Med.

[2] Lawson RA (2017). Stability of mild cognitive impairment in newly diagnosed Parkinson’s disease. J Neurol Neurosurg Psychiatry.

[3] Marshall GA (2015). Functional Activities Questionnaire items that best discriminate and predict progression from clinically normal to mild cognitive impairment. Curr Alzheimer Res.

[4] Altieri M, Garramone F, Santangelo G (2021). Functional autonomy in dementia of the Alzheimer’s type, mild cognitive impairment, and healthy aging: a meta-analysis. Neurol Sci.

[5] Gold DA (2012). An examination of instrumental activities of daily living assessment in older adults and mild cognitive impairment. J Clin Exp Neuropsychol.

[6] Foster ER (2014). Instrumental activities of daily living performance among people with Parkinson’s disease without dementia. Am J Occup Ther.

[7] Pirogovsky E (2014). Instrumental activities of daily living are impaired in Parkinson’s disease patients with mild cognitive impairment. Neuropsychology.

[8] Becker S (2020). Assessment of cognitive-driven activity of daily living impairment in non-demented Parkinson’s patients. J Neuropsychol.

[9] Glonnegger H (2016). The multiple object test as a performance based tool to assess cognitive driven activity of daily living function in Parkinson’s disease. J Alzheimers Dis.

[10] Pfeffer RI (1982). Measurement of functional activities in older adults in the community. J Gerontol.

[11] Kaur N (2016). Critical appraisal of questionnaires to assess functional impairment in individuals with mild cognitive impairment. Int Psychogeriatr.

[12] Martyr A (2019). The relationship between perceived functional difficulties and the ability to live well with mild-to-moderate dementia: findings from the IDEAL programme. Int J Geriatr Psychiatry.

[13] Becker S et al (2021) Everyday function in Alzheimer’s and Parkinson’s patients with mild cognitive impairment*.* J Alzheimers Dis 79(1):197–209 10.3233/JAD-200256 33216023

[14] Christ JB (2013). How precise are activities of daily living scales for the diagnosis of Parkinson’s disease dementia?. A pilot study Parkinsonism Relat Disord.

[15] Shulman LM (2006). Subjective report versus objective measurement of activities of daily living in Parkinson’s disease. Mov Disord.

[16] Liepelt-Scarfone I et al Clinical characteristics with an impact on ADL functions of PD patients with cognitive impairment indicative of dementia*.* PLoS One, 2013. 8(12): p. e82902. 10.1371/journal.pone.0082902 PMC3857297 24349393

[17] Copeland JN (2016). Accuracy of patient and care partner identification of cognitive impairments in Parkinson’s disease-mild cognitive impairment. Mov Disord.

[18] Hipp G (2018). The Luxembourg Parkinson’s Study: a comprehensive approach for stratification and early diagnosis. Front Aging Neurosci.

[19] Szewczyk-Krolikowski K (2014). The influence of age and gender on motor and non-motor features of early Parkinson’s disease: initial findings from the Oxford Parkinson Disease Center (OPDC) discovery cohort. Parkinsonism Relat Disord.

[20] Goetz CG (2008). Movement Disorder Society-sponsored revision of the Unified Parkinson’s Disease Rating Scale (MDS-UPDRS): scale presentation and clinimetric testing results. Mov Disord.

[21] Beck AT (1961). An inventory for measuring depression. Arch Gen Psychiatry.

[22] Nasreddine ZS (2005). The Montreal Cognitive Assessment, MoCA: a brief screening tool for mild cognitive impairment. J Am Geriatr Soc.

[23] Litvan I (2012). Diagnostic criteria for mild cognitive impairment in Parkinson’s disease: Movement Disorder Society Task Force guidelines. Mov Disord.

[24] Bezdicek O (2016). Toward the processing speed theory of activities of daily living in healthy aging: normative data of the Functional Activities Questionnaire. Aging Clin Exp Res.

[25] Lawson RA (2014). Severity of mild cognitive impairment in early Parkinson’s disease contributes to poorer quality of life. Parkinsonism Relat Disord.

[26] Simundic AM (2009). Measures of diagnostic accuracy: basic definitions. EJIFCC.

[27] Choi SM (2019). Analysis of characteristics affecting instrumental activities of daily living in Parkinson’s disease patients without dementia. Neurol Sci.

[28] Skinner JW (2015). Execution of activities of daily living in persons with Parkinson disease. Med Sci Sports Exerc.

[29] Lee WJ (2014). Comparison of activities of daily living impairments in Parkinson’s disease patients as defined by the Pill Questionnaire and assessments by neurologists. J Neurol Neurosurg Psychiatry.

[30] Ueno T (2020). Assessing the relationship between non-motor symptoms and health-related quality of life in Parkinson’s disease: a retrospective observational cohort study. Neurol Sci.

[31] Leroi I (2012). Cognitive impairment in Parkinson disease: impact on quality of life, disability, and caregiver burden. J Geriatr Psychiatry Neurol.

[32] Rosenthal E (2010). Association between cognition and function in patients with Parkinson disease with and without dementia. Mov Disord.

[33] Bloomfield J, Woods D, Ludington J (2016). Self-awareness of memory impairment in Parkinson’s disease: a review of the literature. Working with Older People.

[34] Martinez-Martin P (2008). Burden, perceived health status, and mood among caregivers of Parkinson’s disease patients. Mov Disord.

[35] Tew EH et al Quality of life in Parkinson’s disease caregivers: the contribution of personality traits*.* Biomed Res Int, 2013. 2013: p. 151872. 10.1155/2013/151872 PMC3760184 24024183

[36] Bjornestad A (2016). Loss of independence in early Parkinson disease: a 5-year population-based incident cohort study. Neurology.

[37] Tappen RM, Rosselli M, Engstrom G (2010). Evaluation of the Functional Activities Questionnaire (FAQ) in cognitive screening across four American ethnic groups. Clin Neuropsychol.

[38] Ng A (2015). Influence of depression in mild Parkinson’s disease on longitudinal motor and cognitive function. Parkinsonism Relat Disord.

