# Supplementary material for: Quantifying activities of daily living impairment in Parkinson’s disease using the Functional Activities Questionnaire
Source: Neurol Sci. 2021 Jun 10;43(2):1047–54. doi: 10.1007/s10072-021-05365-1 (PMC8789696; doi:10.1007/s10072-021-05365-1)
Supplement: Supplementary file 1 — Supplementary file1 (DOCX 20.0 KB) [file 10072_2021_5365_MOESM1_ESM.docx]

Supplementary Table 1. *Demographics of 453 Informant Ratings split according to different FAQ cut-offs*

|  | FAQ < 1  *N*=221 | FAQ ≥ 1  *N*=232 | *p*-value | FAQ < 3  *N*=312 | FAQ ≥ 3  *N*=141 | *p*-value | FAQ < 5  *N*=361 | FAQ ≥ 5  *N*=92 | *p*-value |
| --- | --- | --- | --- | --- | --- | --- | --- | --- | --- |
| Male Sex: n (%) | 132 (59.7) | 168 (72.4) | **0.005** | 199 (63.8) | 101 (71.6) | 0.11 | 234 (64.8) | 66 (71.7) | 0.22 |
| Age (years) | 66.87 (47.47-85.40) | 70.25 (46.52-89.28) | **<0.001** | 67.44 (47.36-89.28) | 71.71 (46.52-87.96) | **<0.001** | 67.92 (46.66-89.28) | 72.58 (46.52-87.96) | **<0.001** |
| Education Years | 14 (5-25) | 13 (5-31) | 0.11 | 13 (5-31) | 13 (5-25) | 0.50 | 13 (5-31) | 12 (6-25) | **0.02** |
| Disease Duration Years | 4.37 (0-26) | 6 (0-23) | **0.001** | 4.79 (0-26) | 6.65 (0-23) | **<0.001** | 5 (0-26) | 6.87 (0-22) | **0.001** |
| UPDRS-III Total Score | 27 (7-62) | 36 (1-81) | **<0.001** | 27.5 (1-63) | 38 (3-81) | **<0.001** | 29 (1-75) | 39 (3-81) | **<0.001** |
| BDI-II Total Score | 6 (0-19) | 9 (0-19) | **<0.001** | 6 (0-19) | 11 (0-19) | **<0.001** | 7 (0-19) | 11 (0-19) | **<0.001** |
| MoCA Total Score | 27 (18-30) | 26 (18-30) | **0.004** | 27 (18-30) | 25 (18-30) | **0.001** | 27 (18-30) | 26 (18-30) | **0.003** |

Results are expressed as *Median* (Range) except where noted; Boldface indicates statistically significant values

BDI-II, Beck Depression Inventory-II; FAQ, Functional Activities Questionnaire; MoCA, Montreal Cognitive Assessment; UPDRS-III, Unified Parkinson’s Disease Rating Scale-Part III
