# Supplementary material for: Quantifying activities of daily living impairment in Parkinson’s disease using the Functional Activities Questionnaire
Source: Neurol Sci. 2021 Jun 10;43(2):1047–54. doi: 10.1007/s10072-021-05365-1 (PMC8789696; doi:10.1007/s10072-021-05365-1)
Supplement: Supplementary file 2 — Supplementary file2 (DOCX 16.0 KB) [file 10072_2021_5365_MOESM2_ESM.docx]

Supplementary Table 2. *Demographics of 136 Self Ratings split according to different FAQ cut-offs*

|  | FAQ < 1  *N*=80 | FAQ ≥ 1  *N*=56 | *p*-value | FAQ < 3  *N*=111 | FAQ ≥ 3  *N*=25 | *p*-value | FAQ < 5  *N*=120 | FAQ ≥ 5  *N*=16 | *p*-value |
| --- | --- | --- | --- | --- | --- | --- | --- | --- | --- |
| Male Sex: n (%) | 48 (60) | 40 (71.4) | 0.20 | 70 (63.1) | 18 (72) | 0.49 | 76 (63.3) | 12 (75) | 0.42 |
| Age (years) | 66.35 (48.07-89.93) | 68.44 (50.18-86.39) | 0.12 | 66.51 (48.07-89.93) | 68.93 (50.84-86.39) | 0.27 | 66.34 (48.07-89.93) | 71.13 (50.84-86.39) | 0.06 |
| Education Years | 13 (7-21) | 13 (7-19) | 0.37 | 13 (7-21) | 12 (7-18) | 0.10 | 13 (7-21) | 13 (9-16) | 0.41 |
| Disease Duration Years | 4 (0.08-13.37) | 5.15 (0-31) | 0.12 | 4 (0-13.37) | 6.65 (0.91-31) | **0.02** | 4.14 (0-13.37) | 5.88 (0.91-31) | 0.15 |
| UPDRS-III Total Score | 22 (5-53) | 29.5 (4-81) | **0.001** | 24 (4-53) | 37 (11-81) | **<0.001** | 25 (4-53) | 33.5 (11-81) | **0.002** |
| BDI-II Total Score | 5.5 (0-19) | 7 (1-18) | **0.05** | 5 (0-19) | 10 (2-18) | **0.002** | 6 (0-19) | 9 (2-18) | 0.05 |
| MoCA Total Score | 27 (18-30) | 25 (18-30) | **0.03** | 27 (18-30) | 24 (20-28) | **<0.001** | 26.5 (18-30) | 23.5 (21-28) | **0.001** |

Results are expressed as *Median* (Range) except where noted; Boldface indicates statistically significant values

BDI-II, Beck Depression Inventory-II; FAQ, Functional Activities Questionnaire; MoCA, Montreal Cognitive Assessment; UPDRS-III, Unified Parkinson’s Disease Rating Scale-Part III
